# Validation of periprosthetic joint infection after total knee arthroplasty: a cohort study on 423 cases from the Norwegian Arthroplasty Register

**DOI:** 10.2340/17453674.2026.46073

**Published:** 2026-06-10

**Authors:** Martin MOSSIGE, Olav LUTRO, Anne Marie FENSTAD, Tesfaye Hordofa LETA, Trond BRUUN, Ove FURNES, Håvard DALE

**Affiliations:** 1 The Norwegian Arthroplasty Register, Department of Orthopaedic Surgery, Haukeland University Hospital, Bergen;; 2 Department of Medicine, Stavanger University Hospital, Stavanger; 3 Department of Medicine, Haukeland University Hospital, Bergen; 4 Department of Clinical Medicine, University of Bergen, Bergen; 5 Department of Nursing and Health promotion, Oslo Metropolitan University, Oslo, Norway

## Abstract

**Background and purpose:**

Periprosthetic joint infection (PJI) is a serious complication following total knee arthroplasty (TKA). Accurate reporting is essential for quality development and research based on data in the Norwegian Arthroplasty Register (NAR). Our study aimed to validate Norwegian orthopedic surgeons’ reporting of reoperations for PJI.

**Methods:**

We assessed detailed clinical, biochemical, and microbiological data on 423 patients reported to the NAR for reoperations after primary TKA performed in Western Norway in the period from 2010 to 2023. We used the Musculoskeletal Infection Society (MSIS) definition of PJI. For each cause of reoperation (including causes that could mimic PJI, such as aseptic loosening, prolonged wound drainage, and pain alone), we determined the sensitivity, specificity, negative and positive predictive values, and accuracy.

**Results:**

Of 423 reoperations, 170 were reported reoperations due to PJI. After validation, 94% (159 of 170) were confirmed to be due to PJI. Furthermore, 5% (13 of 253) of cases reported as reoperated due to causes other than deep infection were validated as PJI. The sensitivity, specificity, and accuracy for PJI reporting were 92%, 96%, and 94%, respectively. Accuracy for aseptic loosening, prolonged wound drainage, and pain alone was 97%, 94%, and 98%, respectively.

**Conclusion:**

The accuracy of reporting of reoperations due to PJI is high, thereby affirming the quality of reoperation data concerning PJI submitted to the NAR. Our findings support the use of NAR data in quality development and research.

ORCIDs, if available, can be found on the article page (https://www.actaorthop.org/actao/)

Periprosthetic joint infection (PJI) after total knee arthroplasty (TKA) is a serious complication, with subsequent potential loss of function, and increased morbidity and mortality [1]. Moreover, PJI is associated with high healthcare costs [2]. As the prevalence of TKAs is prospected to increase in the future due to an aging population and increased prosthesis longevity, reducing risk of PJI is of significant importance [3].

The national arthroplasty registers are key drivers in this continuous improvement, owing to high completeness of reporting and long-term follow up on a nationwide cohort [4-6]. However, there have been suggestions of under- and misreporting of PJI to the arthroplasty registers [7-9]. Since the cause of reoperation is to be reported immediately after surgery, based on pre- and intraoperative assessment, incorrect reporting of the cause of reoperation may arise. The lack of results from bacterial cultures is the most obvious limitation, as the samples are cultured over several days postoperatively [7-9]. These concerns regarding the accuracy of the reporting of PJI due to misdiagnosis at the time of reoperation challenge the validity of register data.

The primary objective of our study was to assess the accuracy—and thereby the validity—of the reported cause of reoperation in the NAR due to PJI. Our secondary objective was to evaluate the validity of reported aseptic loosening, prolonged wound drainage, and pain alone, as all these causes of reoperation may resemble PJI clinically.

## Methods

### Study design

The study is reported according to the STARD statement [10].

### Materials/setting

The NAR collects data on primary TKA, and any subsequent reoperations of the prosthesis are linked to the primary TKA by national identity number and laterality. Immediately after performing primary TKA, or a subsequent reoperation, the surgeon reports to the NAR using a standardized form, which includes data such as cause and type of TKA, as well as patient characteristics, procedure, and implant. The form is available in the Supplementary material.

The completeness of reporting of primary TKA to the NAR was 96.2% and 91.5% for reoperations of TKA from 2010 to 2023. For Western Norway, the corresponding completeness of reporting in the study period was 95.1% for primary TKA and 91.8% for reoperations of TKA [4].

In this study, all hospitals in the Western Norwegian Regional Health Authority (Helse Vest RHF) performing reoperations on TKA were examined. This public health trust is 1 of 4 Norwegian regional health trusts, covering 1/5 of the country’s total population [11]. In the study period, 12 public hospitals performed primary and revision TKA. Reoperations are performed by public hospitals exclusively. The hospitals in the health trust use the same electronic health record system, providing complete access to clinical assessments based on radiology, laboratory and surgery reports.

### Data

The reported causes of reoperation included were deep infection, aseptic loosening, prolonged wound drainage, and pain alone. These causes were assumed to deem the highest likelihood to hide PJIs. Several causes of reoperation can be reported to the NAR. All TKAs reoperated with the removal and/or exchange of components, as well as with surgical debridement, antibiotics, and implant retention, with or without exchange of modular parts (DAIR), were included. The use of the term “deep infection” has a long history in the registers. To prevent misunderstandings, the term “deep infection” is used when reported as such by the surgeon, while the term “PJI” is reserved for use after validation.

### Definitions

The review of the electronic patient journal for the identified TKA patients included surgical reports, microbiological results of blood, synovial fluid, and intraoperative samples, and other journal entries documenting pre-, intra-, and postoperative clinical assessments. A standardized form was used to systemize the data.

We applied the definition of PJI provided by the Musculoskeletal Infection Society (MSIS) [12]. 2 positive cultures of phenotypically identical bacteria or the presence of a sinus tract communicating with the joint were considered as PJI. As the MSIS definition minor criteria do not discriminate between high and low virulence species when only 1 positive culture exists, we considered the species of bacteria in addition. With high virulence bacteria such as Staphylococcus aureus and Streptococci species (spp.), only 1 positive culture was sufficient to conclude the presence of PJI, in accordance with the Workgroup Convened by the Musculoskeletal Infection Society [12]. Single findings of low virulence bacteria present, such as coagulase negative Staphylococcus spp. and Cutibacterium spp., were considered as a likely contamination, and not PJI. In these cases, we considered biochemical markers such as CRP and/or ESR, the presence of purulence, and the use of alpha-defensin rapid tests if available to decide whether PJI or not. Use of other markers in synovial fluid was minimal, as was the use of histopathological samples. If no tissue samples were collected intraoperatively, the validated cause of reoperation was based on preoperative bacterial samples (joint aspirations), clinical description of symptoms, results from blood tests, treatment, and clinical course.

According to the guidelines for treating PJI in Norwegian hospitals, a minimum of 5 periprosthetic tissue samples, with the potential addition of deep perioperative bacterial swabs, implant parts, and/or synovial fluid is recommended [13]. The recommended incubation time is at least 8 days.

### Statistics

To determine the validity of the reported data, the sensitivity, specificity, positive and negative predictive values, and accuracy were calculated (Table 1).

**Table 1:** Cross-tabulation for the calculation of true and false positive and negatives, sensitivity, specificity, positive and negative predictive values, and accuracy of reporting

Validated cause of reoperation	Reported cause of reoperation		
	Deep infection	Not infection	
Periprosthetic joint infection	True positive (TP)	False negative (FN)	Sensitivity TP/(TP+FN)
Not infection**^a^**	False positive (FP)	True negative (TN)	Specificity TN/(TN+FP)
	Positive predictive value PPV = TP/(TP+FP)	Negative predictive value NPV = TN/(TN+FN)	Accuracy (TP+TN)/(TP+TN+FP+FN)

a Not infection includes reoperation due to aseptic loosening, prolonged wound drainage, and pain only.

The sensitivity reveals the probability of the surgeon reporting true positive PJI in the case of a PJI, based on pre- and intraoperative data. The specificity reveals the probability of the surgeon reporting true negative PJI, when the TKA was not infected. The positive predictive value illustrates the probability that there is infection present when reported by the surgeon. Inversely, the negative predictive value shows the probability of the TKA being infection free when reported as such. Lastly, the accuracy indicates the probability of agreement between the surgeons’ reported cause of reoperation and the validated cause. 95% confidence intervals for proportions were calculated using the Wilson score interval.

### Ethics, data sharing plan, funding, use of AI, and disclosures

The study was fully financed by the NAR, and approved by the Regional Ethics Committee, REK 209074 (2021, revised 2024). The data collection and the study were performed with patient consent and in accordance with Norwegian and EU data protection rules. AI tools were not used. One of the authors reported a disclosure not relevant to the study. Complete disclosure of interest forms according to ICMJE are available on the article page, doi: 10.2340/17453674.2026.46073

## Results

From January 1, 2010, to December 31, 2023, 88,769 primary TKAs were reported to the NAR. Inclusion and exclusion of cases is presented in the Figure. Primary TKAs performed in health trusts other than the Western Norwegian Regional Health Authority were excluded (n = 72,016), as well as non-reoperated primary TKAs (n = 16,034). Furthermore, TKAs reoperated on due to causes other than those possibly due to PJI were excluded (n = 287), and so were reoperations performed in health trusts other than Helse Vest RHF (n = 9). This results in 423 TKA reoperations eligible for validation for possible PJI. The study population did not differ from those operated on in other regions (Table 2).

**Table 2:** Overview of primary total knee arthroplasty (TKA) and first reoperations in Western Norway compared with the rest of Norway as reported to the Norwegian Arthroplasty Register from 2010 to 2023 in terms of completeness, demographics, primary indication, cause of reoperation, endpoint, and time of follow-up

Item	Western Norway		The rest of Norway	
	Primary TKA	First reoperation of TKA	Primary TKA	First reoperation of TKA
Number of TKA, n (%)	16,753	719 (4.3) **^a^**	72,016	3,307 (4.6) **^a^**
Completeness of reporting, %	95.1	91.8	96.5	91.5
Mean age, years	68.2	65.5	68.1	64.8
Sex, %				
Female	56	57	58	55
Male	44	43	42	45
ASA class, %				
1	10	12	11	13
2	72	67	67	63
≥3	16	20	21	22
Missing	2	1	2	2
Indication for primary TKA, %				
Osteoarthritis	91	86	90	86
Other	9	14	10	14
Causes of first reoperation, n (%)				
Prolonged wound drainage		12 (0.1)		37 (0.1)
Infection		170 (1.0)		833 (1.2)
Aseptic loosening		125 (0.7)		489 (0.7)
Pain only		116 (0.7)		703 (1.0)
Other		296 (1.8)		1,245 (1.7)
Median time to first reoperation (IQR)				
Prolonged wound drainage, days		18 (11–31)		18 (11–31)
Infection, days		82 (22–552)		113 (22–623)
Aseptic loosening, years		2.9 (1.1–4.7)		2.7 (1.2–5.0)
Pain only, years		2.8 (1.5–4.4)		2.4 (1.4–4.0)
Other, years		2.4 (1.1–5.3)		2.2 (0.9–4.3)
Median years of follow up (IQR)	5.2 (2.4–8.3)		5.2 (2.3–8.6)	

a Prevalence

IQR = interquartile range.

**Figure:**
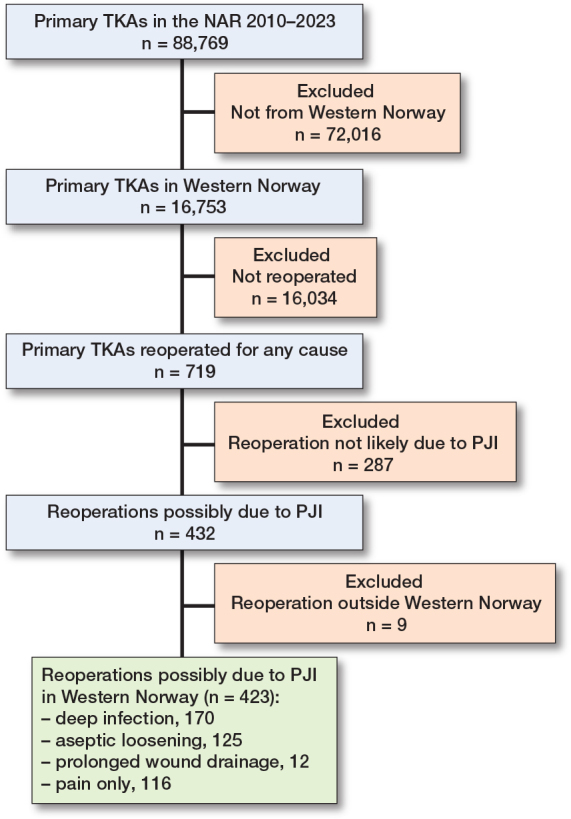
Flowchart presenting inclusion and exclusion of total knee arthroplasty (TKA) in the study. NAR = Norwegian Arthroplasty Register, PJI = periprosthetic joint infection.

The included TKA reoperations had incidence, reporting completeness, and patient characteristics comparable to the national TKA population. We examined 423 reoperations, in 411 patients, that may have been due to PJI, in which 170 were reported as being due to deep infection, 125 for aseptic loosening, 12 for prolonged wound drainage, and 116 for pain alone (Table 3). After validation, there were 172 cases of PJI, 124 cases of aseptic loosening, 11 cases of prolonged wound drainage, and 116 reoperations for pain alone.

**Table 3:**
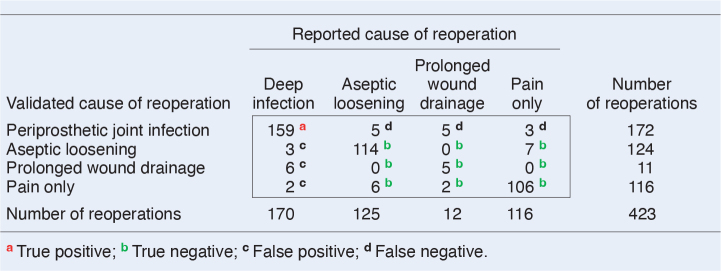
Overview over reported and validated cause of first reoperation following primary total knee arthroplasty in Western Norway, 2010–2023

Sensitivity, specificity, accuracy, and positive and negative predictive values are presented in Table 4. Of the reported 170 reoperations for deep infection, 159 were validated to be PJI. An additional 13 PJI cases had been misdiagnosed and were reported as being reoperated on due to one of the other causes, resulting in a total of 172/423 validated as PJI. This resulted in a sensitivity of 92% and specificity of 96%. The accuracy of reporting was 94%. When TKAs were reoperated on for suspected PJI, aseptic loosening, prolonged wound drainage, or pain alone, surgeons correctly identified the diagnosis in 94% of cases.

**Table 4:** Calculated true and false positives and negatives, positive and negative predictive values, sensitivity, specificity, and accuracy from reported reoperations to the Norwegian Arthroplasty Register, 2010–2023

Other causes of revision	Reoperated for periprosthetic joint infection								
	True positive	True negative	False positive	False negative	PPV, % (CI)	NPV, % (CI)	Sensitivity % (CI)	Specificity % (CI)	Accuracy % (CI)
Aseptic loosening, prolonged wound drainage, or pain only	159	240	11	13	94 (89–96)	95 (91–97)	92 (87–96)	96 (93–98)	94 (92–97)
Aseptic loosening	159	114	3	5	98 (95–99)	96 (91–98)	97 (93–99)	97 (93–99)	97 (94–99)
Prolonged wound drainage	159	5	6	5	96 (92–98)	50 (25–76)	97 (93–99)	45 (21–72)	94 (89–96)
Pain only	159	106	2	3	99 (96–100)	97 (92–99)	98 (95–99)	98 (93–99)	98 (96–99)

PPV = positive predictive value, NPV = negative predictive value, CI = 95% confidence interval.

## Discussion

We aimed to evaluate the validity of Norwegian surgeons’ reporting of PJI to the NAR following the first reoperation after primary TKA. We identified the accuracy of reporting PJI, aseptic loosening, prolonged wound drainage, and pain alone as 94%. The sensitivity and specificity of reported PJI were high, being 92% and 96% respectively.

Regarding PJI, our findings are higher than a previous study from the NAR on total hip arthroplasties (THA) [14]. Lutro et al. found a somewhat lower accuracy of 87% reported for THA reoperations, with a sensitivity of PJI of 80% and a specificity of 94%. The slightly better accuracy for TKA is owed in part to the relatively greater number of reoperations due to pain alone and fewer reoperations reported for prolonged wound drainage in TKA, in comparison with THA. Even so, the reporting accuracy for reoperations after THA and TKA is good, with limited misclassification, thereby confirming the robustness of the NAR data.

Two Nordic studies have also validated reoperations for PJI in the Danish and Swedish arthroplasty registers, for THA and TKA, with the use of microbiological data [15-17]. However, these studies focused on a quantitative validation of reporting against an epidemiological national microbiological database and a prescribed drugs register. We have performed a detailed, qualitative validation of reporting of reoperations for PJI to the NAR, with in-depth assessment of the individual TKA patient. Thus, we did not compare our findings with national databases or assess the data quantitatively, while the authors of the Nordic studies did not perform a qualitative validation while taking all individual clinical assessments into account. As the completeness of reporting is not 100% in the NAR, we cannot exclude the possibility that the true sensitivity among the population is lower than found in this study, as there may have been infections that have not been reported rather than being misclassified.

125 cases were reported as reoperated on due to aseptic loosening, of which only 5 were reclassified as PJIs after validation, comprising an accuracy of 97%. This is in line with the validation on THA from the NAR [14], where they found a minor underestimation of PJI in the cases of reported reoperation for aseptic loosening. Other studies on occult infections being diagnosed as aseptic loosening in TKA have also concluded that there is an underestimation of PJI incidence, and that PJI may resemble aseptic loosening [18].

Low-grade infections may be difficult to diagnose before conclusive microbiology reports are available. Clinical signs of PJI may be sparse, and the presence of low-virulence bacteria may produce conflicting diagnostic test results. Moreover, chronic low-grade TKA infections often have a non-acute onset [2].

Consequently, as a recent study links the presence of subclinical biofilms produced by these bacteria to osteolytic loosening, it is essential to collect tissue samples during reoperation for aseptic loosening [19]. With low bacterial load and biofilm-bound bacteria often being associated with low-virulence bacteria, a negative joint aspiration culture in combination with few symptoms may lead to the misdiagnosis of aseptic loosening, when in fact PJI is the root cause [2] . Intraoperative bacterial sampling may therefore increase the accuracy of differentiating aseptic and septic loosening.

116 out of 423 reoperations were done due to pain alone, a significantly higher proportion than after THA [14]. A negligible 3 of these reoperations were caused by PJI, confirming that PJI rarely presents with pain only after TKA.

However, low-grade infections may present with pain and arthrofibrosis as the most prominent or only symptoms [2]. Only if the surgeon is aware of this and collects intraoperative bacterial samples may the distinction between pain alone and PJI be made and treated correctly.

The accuracy in distinguishing pain alone from PJI and aseptic loosening is high (98%). In many of the reoperations for pain alone, PJI or aseptic loosening may have been ruled out as the cause by joint aspirations, blood samples, radiology, and nuclear imaging, in addition to clinical examination, before being reoperated. This is in line with studies showing a specificity between 87% and 100% in disproving PJI on the basis of joint aspirations [20,21]. Combined with a reported high specificity of disproving aseptic loosening from radiological imaging [22], the validated cause of reoperation and reported cause remain identical for a majority of the reoperations for pain alone. The accuracy is therefore high.

In total, 5 out of 12 reported reoperations for prolonged wound drainage were in fact PJIs. Some of the cases also had superficial surgical site infections (SSI) with positive preoperative bacterial swabs, but negative intraoperative bacterial samples. We found a low number of reported wound problems among the reoperations. As the cause of reoperation is reported immediately after surgery, prolonged wound drainage may be the correct diagnosis to report if the PJI status is unknown at the time of surgery. However, wound problems and superficial SSI are strongly associated with PJI [23,24] and appear to be reported correctly as PJI by Norwegian surgeons, hence the relatively low number of reported reoperations for prolonged wound drainage.

In the previous validation study on THA, 22 out of 37 (60%) reoperations for prolonged wound drainage after THA were validated to PJI [14], compared with the lower 5 out of 12 (42%) reoperations in this study. This difference in misclassification may be attributed to anatomical differences in the soft tissue envelope between the hip and the knee. The proximity of the TKA to the skin may increase the risk of PJI when prolonged wound drainage is present. Based on this knowledge, our findings suggest that surgeons more often suspect and report PJI as cause of reoperation after primary TKA compared with THA, given prolonged wound drainage.

### Strengths

The data quality of the NAR is high, owing to high coverage of reporting hospitals and completeness of reporting. The patient and procedure characteristics of the TKA population of Western Norway were considered representative of the general Norwegian population. The external validity of the present study is therefore considered to be good.

We identified all the patients from the same electronic journal system. This includes all documented clinical, biochemical, and microbiological data. Our validation may thus reveal PJI not identified by assessing single sources, making the validation as complete as possible.

### Limitations

The quantity of reporting was not assessed. However, the coverage of hospitals was 100% and the completeness of reporting was 95.1% for primary TKA and 91.8% for reoperations, indicating minor under-reporting. We may have underestimated the number of PJI after TKA in this study, owing to the lack of tissue samples collected in several reoperations for aseptic loosening or pain alone, or the lack of information on the minor criteria of the MSIS definition of PJI, such as CRP/ESR and synovial fluid analyses. The Norwegian guidelines recommend collecting a minimum of 5 tissue samples in reoperations for suspected PJI [13]. These guidelines do not apply to reoperations for aseptic causes. In addition, the MSIS definition confirms the presence of an infection when 2 or more phenotypically identical bacterial samples are present, or when a set of minor criteria are fulfilled [25]. We have not been able to examine these minor criteria systematically, as tests such as alpha-defensin or calprotectin in synovial fluid, or histopathology samples, are not used routinely in Norwegian hospitals.

Lastly, reporting to the NAR may be subject to several biases. As the NAR presents statistics from different hospitals, some surgeons may feel obliged to report a more favorable cause of reoperation than PJI, so-called “gaming”, when in doubt. Also, a few of the cases present with lacking documentation in the patient journal, thus validation proved difficult.

### Conclusion

We found that the sensitivity and specificity of reported PJI were high, being 92% and 96%, respectively, and an accuracy of 94%.

*In perspective*, we believe that PJI is a valid endpoint to be used in quality development and research studies.

## Notes

MM was responsible for data collection and writing the manuscript. MM and HD performed the analyses and validations. AMF extracted the dataset from the NAR. THL contributed to the data collection. All authors contributed to critical review of the manuscript.

Handling co-editors: Keijo Mäkelä and Philippe Wagner

*Acta* thanks Martin Lindberg-Larsen and Anna Stefánsdóttir for help with peer review of this manuscript.
